# High Stocking Density and Food Deprivation Increase Brain Monoaminergic Activity in Gilthead Sea Bream (*Sparus aurata*)

**DOI:** 10.3390/ani11061503

**Published:** 2021-05-22

**Authors:** Marcos Antonio López-Patiño, Arleta Krystyna Skrzynska, Fatemeh Naderi, Juan Miguel Mancera, Jesús Manuel Míguez, Juan Antonio Martos-Sitcha

**Affiliations:** 1 Laboratorio de Fisioloxía Animal, Departamento de Bioloxía Funcional e Ciencias da Saúde, Facultade de Bioloxía and Centro de Investigación Mariña, Universidad de Vigo, 36310 Vigo, Spain; mlopezpat@uvigo.es (M.A.L.-P.); Fatemeh.Naderi79@uvigo.es (F.N.); jmmiguez@uvigo.es (J.M.M.); 2 Department of Biology, Faculty of Marine and Environmental Sciences, Campus de Excelencia Internacional del Mar (CEI·MAR), Instituto Universitario de Investigación Marina (INMAR), University of Cádiz, 11519 Puerto Real, Spain; arleta.skrzynska@uca.es (A.K.S.); juanmiguel.mancera@uca.es (J.M.M.)

**Keywords:** brain monoamines, cortisol, dopamine, serotonin, stress, teleost, welfare

## Abstract

**Simple Summary:**

Farmed fish must cope with different stressors during aquaculture procedures, such as high densities, fasting, transport, or air exposure during handling. The severity and timing of these stressors can produce important imbalances in the overall status of the animals, triggering several endocrine and physiological players. In this study, gilthead sea bream (*Sparus aurata*) juveniles were assigned to four experimental conditions: (1) fed at a low stocking density (LSD-F, 4 kg·m^−3^); (2) fed at a high stocking density (HSD-F, 40 kg·m^−3^); (3) food-deprived at LSD (LSD-FD); and (4) food-deprived at HSD (HSD-FD). This served to evaluate, both at the plasma and central (brain) levels, the role of several hormonal (cortisol and catecholamines) and monoamine (dopaminergic and serotonergic neurotransmitters) functionalities. Our results evidenced chronic stress exposure (i.e., a high stocking density and food deprivation) fallouts in the enhancement of the parameters related to the stress response, where monoaminergic activities in different brain regions served to reorganize the physiological response depending on the challenge applied.

**Abstract:**

In teleosts, brain monoamines (dopamine and serotonin) participate in the early response to different acute stressors. However, little is known regarding their role during chronic stress. In a 2 × 2 factorial design, the influence of a high stocking density (HSD) and/or food deprivation (FD) on the brain monoaminergic activity in gilthead sea bream (*Sparus aurata*) was evaluated. Following a 21-day experimental design, samples from the plasma and brain regions (telencephalon, hypothalamus, and optic tectum) were collected. The dopamine (DA), serotonin (5HT), and their main metabolites, 3,4-dihydroxyphenylacetic acid (DOPAC) and 5 hydroxyindoleacetic acid (5HIAA), contents were HPLC-assessed in brain tissues, and the ratios DOPAC/DA and 5HIAA/5HT were calculated as indicators of enhanced monoaminergic activity. The plasma levels of cortisol and catecholamine were also evaluated. The cortisol levels increased in fish exposed to HSD and normally fed but, also, in all FD groups, whereas the NA levels decreased in LSD-FD animals. Within the brain, the dopaminergic and serotonergic activities in telencephalon and hypothalamus increased in fish subjected to HSD and in the telencephalon of LSD-FD fish. While DA (hypothalamus) and 5HT (telencephalon) increased in the animals submitted to a HSD, food-deprived fish did not show such an increase. Taken together, our results supported the hypothesis of brain monoaminergic activity participating in maintaining and orchestrating the endocrine response to chronic stress in fish.

## 1. Introduction

The typical manipulations in aquaculture, such as handling, transport, crowding, or vaccination, among others, have undesired effects on fish, thus compromising their welfare. As a result, research is needed to elucidate the mechanisms by which these effects occur, which may be useful when implementing specific strategies to mitigate the negative effects of these manipulations. When subjected to a potentially stressful situation, a stress-related physiological response initiates in these animals to achieve a new homeostatic load. Thus, the hypothalamus–sympathetic nervous system–chromaffin (HSC) and the hypothalamus–pituitary–inter-renal (HPI) axes are activated to face the episode of stress [1,2]. As a consequence, the circulating catecholamine and cortisol levels increase, followed by enhanced glucose and lactate levels [3], and multiple alterations at the metabolic and functional stages also occur [4,5].

Cortisol is the main stress-related hormone, which makes it the most important indicator of stress exposure. The HPI axis controls the synthesis of this steroid in teleost fish and takes place within the inter-renal tissue. The altered function of this tissue results in changes of the circulating cortisol, thus interfering with the ability of fish to cope with a stressful situation, which leads to a decreased chance of survival [5]. The cortisol release is modulated by the adrenocorticotropic hormone (Acth) secreted from the pituitary after their stimulation by the corticotropin-releasing factor (Crf), which is released from the hypothalamus [6]. Several inputs can influence this endocrine axis [7]. In turn, brain monoamine neurotransmitters play a key role in controlling a wide range of physiological and behavioral processes. Regarding the dopaminergic system, tyrosine hydroxylase (TH) cell bodies are located in different nuclei of the forebrain, whereas TH-ir fibers are widely distributed in the telencephalon and diencephalon of fish, such as the chiclid *Astatotilapia burtoni* [8] and zebrafish [9]. Among both TH isoforms (*th1* and *th2*), a variation in the gene expression pattern and tissue distribution among teleost lineage has been identified. Accordingly, *th1* is widely distributed in the brain of zebrafish, in which many telencephalic and diencephalic regions express both isoforms, while few regions only express *th2*, such as the hypothalamic dorsal paraventricular organ (PVO) intermediate nucleus [9]. On the other side, regarding the serotonergic system, the presence of serotonin (5-HT) neurons in raphe nuclei appears to be an evolutionary conserved feature among vertebrates and is probably an ancestral character of chordates [10]. Serotonergic neurons are located in raphe nuclei from all major fish clades, thus giving rise to ascending and descending pathways (see reference [11]), and more than 20 different clusters of 5-HT neurons exist in certain fish [12]. In this way, four main populations of raphe 5-HT neurons with different target areas and morphologies have been identified in zebrafish: (i) dorsally located cells projecting to the olfactory bulb and telencephalon, (ii) ventrally located bigger cells projecting to the hypothalamus, (iii) ventrolaterally cells that project in the general area of the migrated nuclei of the posterior tuberculum and (iv) the most caudal population projecting locally in the hindbrain and the spinal cord (see reference [11]).

Regional studies assessing monoamine content changes in the brain are relevant, considering the specific physiological role of each location and neurotransmitter. In this way, monoaminergic systems within the hypothalamus, telencephalon, optic tectum, and brainstem are reported to participate in the stress response in fish [13,14], whereas these and others are also critical for coordinating specific behaviors, such as social interaction [15], avoidance [16], and feeding regulation [17,18]. Thus, noradrenaline (NA), dopamine (DA), and serotonin (5 hydroxytryptamine, 5HT) influence the control and integration of the behavioral and physiological responses in teleost fish [19,20].

Increased dopaminergic and serotonergic activities within the central nervous system participate in stimulating cortisol synthesis and its release into the blood, as reported for other species, such as Senegalese sole [21] or rainbow trout [14]. It is also accepted in the involvement of brain monoamines (DA and 5HT) in the initiation of the response of teleost fish to different acute stressors (chasing, air exposure, etc.), although information regarding the role of these neurotransmitters during chronic stress is scarce [14,22] and, even more, in marine species such as Senegalese sole [21], sea bass [23], and, in a meagre amount, *Argyrosomus regius* [24]. Among them, gilthead sea bream (*Sparus aurata*) is considered one of the main farmed species in Mediterranean and European aquacultures and, also, a well-recognized animal model attending to their overall physiology, nutrition, and metabolism [25].

In this way, the aim of this work was to unravel and go further on the monoaminergic activity of this species in a factorial design (2 × 2) to induce different stress events produced by a high stocking density and food deprivation independently or by their combination in a three-week trial. The working hypothesis is that each brain region studied (the telencephalon, hypothalamus and optic tectum) could orchestrate at the central nervous system the stress response depending on the challenge applied, with their subsequent repercussion on the overall physiology to develop the best strategies to understand and reduce the impact of typical situations in an aquaculture.

## 2. Materials and Methods

### 2.1. Animals and Experimental Conditions 

Gilthead sea breams, *Sparus aurata* (200 ± 20-g body weigh ± SEM), were obtained from the Servicios Centrales de Investigación en Cultivos Marinos (SCI-CM, CASEM, University of Cádiz, Puerto Real, Cádiz, Spain), and all assay procedures were conducted in their experimental facilities (Spanish Operational Code REGA ES11028000312). Animals were distributed in eight 1000-L tanks adjusted to 625 L of water volume to obtain a final stocking density of 4 kg·m^−3^ used as a control during the experimental approach (see below). The tanks were connected to an open system, and fish acclimated for 15 days to these laboratory conditions before any experimental procedure. Lighting conditions were 12-h L:12-h D, and water salinity (38‰) and temperature (19 °C) were constant during the time the experiment lasted. Water pH, NH_3_, and O_2_ levels were monitored at the effluents of all tanks, both during acclimation and during the experimental period, where O_2_ was above 85% saturation (daily measured), pH stable at pH 7.63 ± 0.06, and unionized ammonia remained below 0.02 mg/L (measured twice a week). During that period, a normal feeding pattern was observed, with food consisting of commercial dry pellets (Dibaq-Diproteg SA, Segovia, Spain; Crude Protein: 44%, Crude Fat: 20%, Carbohydrates: 18.1%, Ash: 7.0%, Total Phosphorus: 0.9%, and Digestible Energy: 17.9 MJ/kg) at a daily ration of 1% of their body mass.

Following acclimation, the water depths of each tank were maintained or lifted to obtain the final density for both the experimental stocking conditions. Thus, 4 tanks (*n* = 12 fish/tank) randomly selected were kept at 4 kg·m^−3^, as described above, whereas, in the remaining 4 tanks (*n* = 12 animals/tank), the fish were introduced in a rigid net, and the water depth was adjusted in order to raise the final stocking density of 40 kg·m^−3^ in a final 62.5-L volume. The total water volume capacity of each tank (1000 L) or the rigid nets (150 L) were allowed to increase the water depth weekly to maintain the initial stocking densities, assuming an average specific growth rate (SGR) of around 1.15–1.20%·day^−1^. This protocol was performed according to that previously reported [26,27]. Once the stocking densities were achieved, the animals were subjected to four different experimental conditions in duplicate (2 tanks per treatment): (1) fed at a low (Control) stocking density (LSD-F; 4 kg·m^−3^), (2) fed at a high stocking density (HSD-F, 40 kg·m^−3^), (3) food deprivation and LSD (LSD-FD), and (4) food deprivation and HSD (HSD-FD). Animals from the LSD-F and HSD-F groups were fed with a daily ration of 1% of their average body mass to avoid differences in the feeding protocols among both groups, whereas the food-deprived fish (LSD-FD and HSD-FD) were unfed up to the end of the experiment (21 days). After this period, 6 fish from each experimental tank (*n* = 12 fish/group) were sacrificed and sampled at the end of the experiment (see below). Such an experimental design was previously reported to induce a stress response in this fish species [26,27,28,29].

### 2.2. Sampling 

Fish were deeply anaesthetized by addition of 2-phenoxyethanol to the tank water (1-mL/L seawater). To guarantee a uniform mix of anesthetize, the appropriate volume of 2-phenoxyethanol was previously diluted in 5 L of tank water and, afterwards, added into the fish tank. This procedure was done in the absence of visual contact between the fish and the manipulators, thus minimizing the incidence of the acute stress response. Once anesthetized, individual blood samples were collected. From each animal, 1 mL of blood was collected from the caudal peduncle by using ammonium-heparinized syringes. Then, the plasma was separated after blood centrifugation (5 min at 10,000× *g*, 4 °C), immediately frozen in liquid nitrogen, and stored at −80 °C until assayed for cortisol and catecholamine (adrenaline, A and noradrenaline, NA) concentrations. Immediately after blood collection, the animals were sacrificed by spinal sectioning, and the individual brains were dissected. Individual telencephalon, hypothalamus, and optic tectum were separated and placed into 1.5-mL Eppendorf tubes, frozen in liquid nitrogen, and stored at −80 °C until HPLC-assayed for the monoamine content (dopamine, DA and serotonin, 5HT) and their respective metabolites, named 3,4 dihydroxyphenylacetic acid (DOPAC) and 5 hydroxyindoleacetic acid (5HIAA).

### 2.3. Analytical Methods

#### 2.3.1. Plasma Cortisol and Catecholamine Levels 

The plasma cortisol levels were measured by using a commercially available Enzyme Immunoassay kit (Cayman, Ann Arbor, MI, USA), according to the manufacturer’s indications, with specific modifications for sea bream [30]. The percentage of recovery was 95%, and the inter- and intra-assay coefficients of variation (calculated from the sample duplicates) were 2.93 ± 0.34% and 4.32 ± 0.59%, respectively.

Plasma adrenaline and noradrenaline levels were assessed by high-performance liquid chromatography (HPLC) with electrochemical detection after purification of the plasma by deproteinization followed by solid-phase extraction (SPE), according to that previously reported [31,32]. In brief, individual 100-μL plasma aliquots were deproteinized with 25 μL of 0.6-mol L^–1^ perchloric acid (HClO_4_; Merck, Darmstadt, Germany) and centrifuged (14,000× *g*, 4 min, 4 °C). Supernatants were then neutralized with 25 μL of 1-mol L^–1^ KHCO_3_ (Merck); centrifuged (14,000× *g*, 4 min, 4 °C); and the supernatants diluted to 1 mL in ultrapure water for use in the SPE procedure. Each SPE cartridge (1 mL–100 mg tubes, Discovery DSC-WCX, Supelco, Bellefonte, PA, USA) was conditioned with 1.5-mL ultrapure water at a 5-mL min^–1^ flow rate. Then, the samples were transferred to the conditioned columns at 1 mL min^–1^ and rinsed by 2× the addition of 1 mL of ultrapure water (5 mL min^–1^). Then, catecholamines were eluted from the columns with two 400-μL aliquots of 0.3-mol L^–1^ HClO_4_ (1 mL min^–1^). Twenty milliliters of each eluate were injected into the HPLC system, consisting of a Jasco PU-2080 Plus pump (Jasco Corp., Tokio, Japan), a 5-μm analytical column (Nucleosil C18, 150-mm length × 4.6-mm diameter; Phenomenex, Macclesfield, Cheshire, UK), and an ESA Coulochem II detector (Chelmsford, MA, USA). The detection system included a double-analytical cell (M5011; ThermoFisher Scientific, Waltham, Massachusetts, USA) with the oxidation potentials set at +40 mV (first electrode) and +400 mV (second electrode). The mobile phase, 25-mmol L^−1^ citric acid (Panreac, Barcelona, Spain), 25-mmol L^−1^ Na_2_HPO_4_ (Merck), 25-μmol L^−1^ Na_2_EDTA (Sigma, St Louis, MO, USA), 0.21-mmol L^−1^ sodium 1-octanesulfonate (Fluka, Sigma), and 1% (*v*/*v*) methanol (Panreac), was pH-adjusted to 3.4 with orthophosphoric acid (before methanol addition), filtered (0.20-μm filter, Millipore, Bedford, MA, USA), and degassed by vacuum before use. The analytical run time was 10 min at an isocratic flow rate of 1.3 mL min^−1^ at room temperature. The sample peaks were quantified by comparing the areas under the curve to those of the appropriate standards. The detection limits per injection for catecholamines were 3 pg (NA) and 5 pg (A), with a signal-to-noise ratio of 3. Chromatogram acquisition and integration were performed using ChromNAV version 1.12 software (Jasco Corp., Tokyo, Japan).

#### 2.3.2. Analysis of Brain Monoamines 

High-performance liquid chromatography (HPLC) with electrochemical detection was assessed to evaluate the content of dopamine (DA), 3,4-dihydroxyphenylacetic acid (DOPAC, the main DA oxidative metabolite), serotonin (5-HT), and 5-hydroxyindole-3-acetic acid (5-HIAA, the main 5-HT oxidative metabolite) in different brain regions (the telencephalon, hypothalamus, and optic tectum), according to a previously described study [33]. Individual brain regions were homogenized by ultrasonic disruption in 0.4 mL of the HPLC mobile phase and centrifuged (16,000× *g*, 10 min), and the supernatants diluted in mobile phase 1:3 before the HPLC assay. One aliquot of each supernatant was separated for further quantification of the protein content following the bicinchoninic acid method [34]. The HPLC system was a Jasco PU-2080 Plus pump, a 5-μm reverse-phase analytical column (Kinetex C18 100 Å, 150-mm length × 4.6-mm diameter; Phenomenex) kept in a Jasco CO-4060 column oven at 25 °C, a Jasco AS-2057 autosampler, and an ESA Coulochem II detector. The detection system included a double-analytical cell (M5011) with the oxidation potentials set at +40 mV (first electrode) and +340 mV (second electrode). The mobile phase (63.9 mol·L^−1^ NaH_2_PO_4_, 0.1 mol·L^−1^ Na_2_EDTA, 0.80 mol·L^−1^ sodium 1-octanesulfonate, and 14% (*v*/*v*) methanol) was pH-adjusted to 2.85 with ortho-phosphoric acid. The mobile phase was filtered (0.20-μm filter, Millipore) and degassed before use. The analytical run time was 12 min at an isocratic flow rate of 1.0 mL·min^−1^. Sample peaks were quantified by comparing areas under the curve to those of the appropriate standards. The ChromNAV software version 1.12 (Jasco Corp.) was used for chromatogram acquisition and integration.

### 2.4. Statistics

Results are shown as the mean ± standard error of the mean (mean ± SEM). After assessing the homogeneity of the variance and normality, the statistical analysis was performed by using a two-way ANOVA test with the “feeding condition” and “stocking density” as the main factors. When a significant effect was identified within a factor, post-hoc comparisons were assessed by using Tukey’s test. A comparison of the duplicate tanks for all parameters was also performed with a Student’s *t*-test to discard any tank effect. *p* < 0.05 was set as the significance level. All tests were performed using the GraphPad Prism^®^ (v.5.0b) (San Diego, CA, USA) software for Macintosh.

## 3. Results

Nonsignificant differences were found for all the parameters assessed between the replicate tanks. In addition, no mortality, health disturbances, or any alterations in fish behavior were observed in any experimental group. The only differences observed were relative to the growth performances of the fish, denoted by positive (HSD: 1.09 ± 0.08%·day^−1^ or LSD: 1.31 ± 0.17%·day^−1^) or negative (−0.62 ± 0.11%·day^−1^) specific growth rates (SGR) in fed and starved fish, respectively.

### 3.1. Plasma Cortisol, Adrenaline, and Noradrenaline Levels

Data on the plasma cortisol, adrenaline, and noradrenaline are shown in Table 1. 

The cortisol levels in the plasma of fish kept under LSD-F were 4.17 ± 0.67 ng·mL^−1^. The exposition to HSD resulted in a significant increase of this hormone in both fed (27.36 ± 0.67 ng·mL^−1^) and food-deprived fish (23.87 ± 6.61 ng·mL^−1^; *p* < 0.05). In addition, a significant enhancement in this hormone was observed in LSD-FD fish (10.06 ± 2.68 ng·mL^−1^) when compared with the LSD-F group. No variation was observed in this parameter between the different food regimens (F vs. FD) at the HSD. The plasma adrenaline levels in LSD-F fish were 97.56 ± 10.05 nM. The HSD condition increased the adrenaline content in the fed fish (148.74 ± 27.53 nM) with respect to the LSD-F group. Food deprivation did not affect the adrenaline content in sea breams kept at a LSD, relative to the LSD-F fish. In addition, the stocking condition did not significantly affect the adrenaline content in FD sea breams. Finally, the noradrenaline levels in LSD-F fish were 79.78 ± 6.72 nM. The HSD did not affect this content in the fed animals (91.78 ± 13.67 nM) with respect to the LSD-F. However, exposing the LSD-adapted fish to food deprivation significantly increased the circulating noradrenaline (49.03 ± 4.99 nM) relative to the LSD-F group. The feeding condition did not affect the noradrenaline content in the animals maintained under a HSD (HSD-F: 91.78 ± 13.67 nM; HSD-FD: 70.92 ± 24.62 nM).

### 3.2. Brain Serotonergic and Dopaminergic Activity

**Figure 1:**
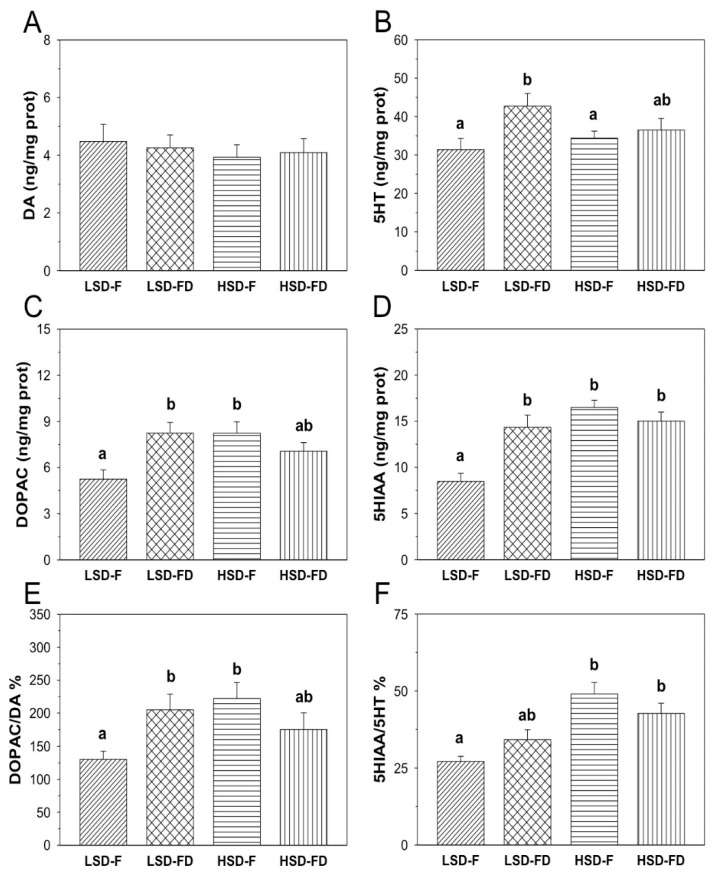
Content of DA (**A**), 5HT (**B**), their main metabolites (DOPAC (**C**), and 5HIAA (**D**)), and the ratios DOPAC/DA (**E**) and 5HIAA/5HT (**F**), in the hypothalamus of *S. aurata* juveniles subjected to feeding (F) or food deprivation (FD) conditions, together with low (LSD) or high (HSD) stocking densities. Different letters indicate significant differences (*p* < 0.05) among the groups. Values represent the average ± S.E.M.; *n* = 12 samples per group.

**Figure 2:**
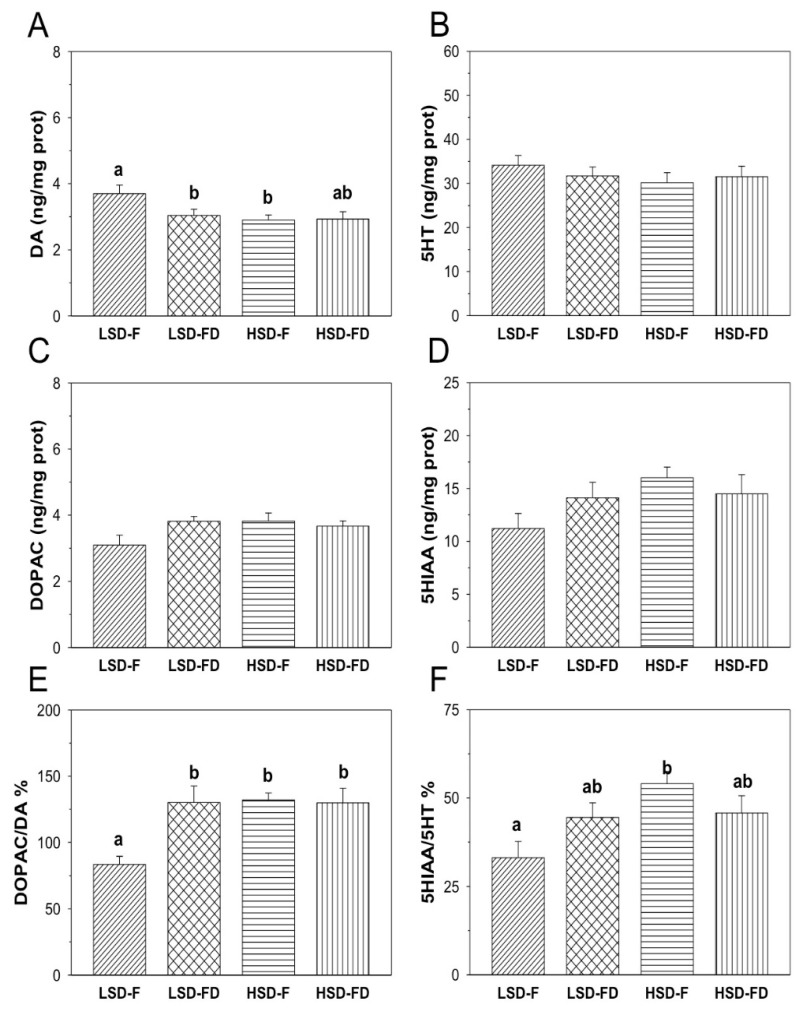
Content of DA (**A**), 5HT (**B**), their main metabolites (DOPAC (**C**), and 5HIAA (**D**)), and the ratios DOPAC/DA (**E**) and 5HIAA/5HT (**F**), in the telencephalon of *S. aurata* juveniles subjected to feeding (F) or food deprivation (FD) conditions, together with low (LSD) or high (HSD) stocking densities. Different letters indicate significant differences (*p* < 0.05) among the groups. Values represent the average ± S.E.M.; *n* = 12 samples per group.

**Figure 3:**
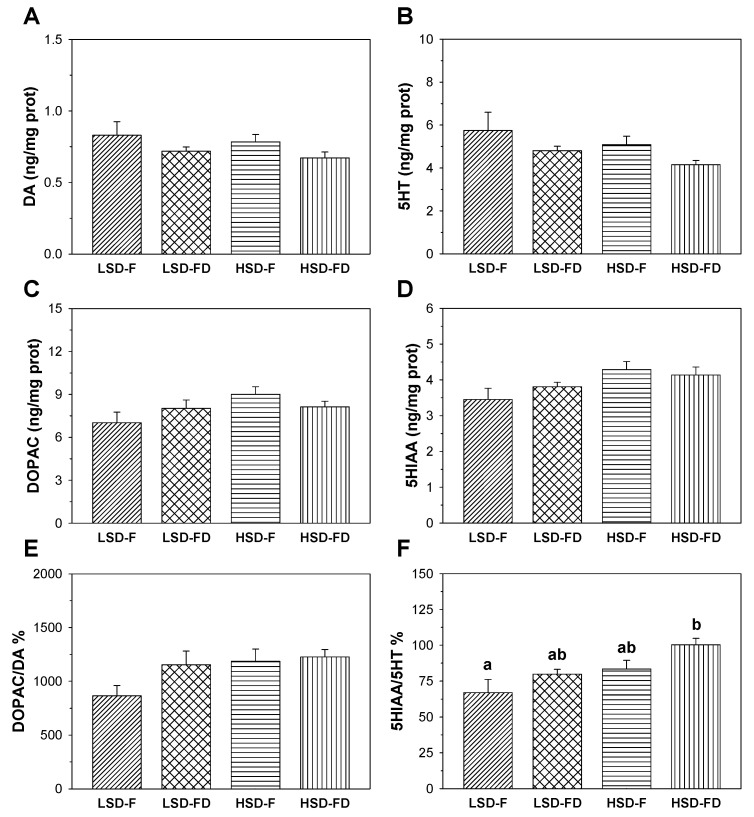
Content of DA (**A**), 5HT (**B**), their main metabolites (DOPAC (**C**), and 5HIAA (**D**)), and the ratios DOPAC/DA (**E**) and 5HIAA/5HT (**F**), in the optic tectum of *S. aurata* juveniles subjected to feeding (F) or food deprivation (FD) conditions, together with low (LSD) or high (HSD) stocking densities. Different letters indicate significant differences (*p* < 0.05) among the groups. Values represent the average + S.E.M.; *n* = 12 samples per group.

The monoamine content (DA and 5HT), their main metabolites (DOPAC and 5HIAA, respectively), and the ratios DOPAC/DA and 5HIAA/5HT in the hypothalamus, telencephalon, and optic tectum of seabream subjected to each experimental condition are shown in Figure 1, Figure 2 and Figure 3.

The hypothalamic DA content in LSD-F fish was 4.48 ± 0.59-ng/mg prot (Figure 1). Neither a high stocking density nor food restriction significantly affected the DA content in this tissue (Figure 1A). On the other side, the DOPAC content (Figure 1C) in the LSD-F group was 5.25 ± 0.60-ng/mg prot. However, the fish under HSD conditions significantly enhanced the DOPAC content in this tissue (HSD-F: 8.23 ± 0.74-ng/mg prot). In addition, the FD animals subjected to LSD also displayed a significant increase of the DOPAC level (8.24 ± 0.69-ng/mg prot). The HSD-FD animals slightly enhanced the DOPAC content, but such an increase did not reach a level of significance. The DOPAC/DA ratio (Figure 1E) displayed identical variations compared to those observed for the DOPAC content. Thus, significantly increased values were found in the HSD-F and LSD-FD groups relative to the LSD-F (*p* < 0.05 respectively), together with a small and nonsignificant increase of the ratio in the HSD-FD group. The hypothalamic 5HT content (Figure 1B) in sea breams from the LSD-F group was 31.41 ± 2.89-ng/mg prot. The HSD condition did not affect the 5HT content in the fed animals and slightly enhanced it in the FD fish (36.50 ± 3.01-ng/mg prot) but did not reach a level of significance relative to the LSD-F group. The animals kept at a LSD under the FD regimen significantly enhanced the 5HT content (42.72 ± 3.31-ng/mg prot) compared to that of the LSD-F animals (*p* < 0.05). The content of 5HIAA (Figure 1D) in the LSD-F group was 8.48 ± 0.87-ng/mg prot. The HSD and FD conditions, alone or together, enhanced the 5HIAA content in the hypothalamus (HSD-F: 16.49 ± 0.79-ng/mg prot; LSD-FD: 14.34 ± 1.32-ng/mg prot; HSD-FD: 15.01 ± 0.98 ng/mg-prot) with respect to the LSD-F animals (*p* < 0.05 each). The 5HIAA/5HT ratio (Figure 1F) in the LSD-F group was 27.11 ± 1.73. However, this ratio enhanced in HSD relative to the LSD-F, whereas the animals kept under LSD but under the FD condition showed a small non-significant increase of the ratio.

In the telencephalon (Figure 2), the DA (Figure 2A) content in the LSD-F group was 3.70 ± 0.26-ng/mg prot. Overall, exposure to the HD and FD conditions resulted in a significant decrease of the DA levels (HSD-F: 2.90 ± 0.15-ng/mg prot; LSD-FD: 3.04 ± 0.18-ng/mg prot) with respect to the LSD-F group (*p* < 0.05 each), whereas in the HSD-FD group, such a decrease did not reach a level of significance. The content of DOPAC (Figure 2C) was not affected by the HSD or/and FD. However, exposing the animals to both experimental conditions significantly enhanced the (*p* < 0.05 for HSD-F, LSD-FD, and HSD-FD) DOPAC/DA ratio (Figure 2E; HSD-F: 131.95 ± 5.24; LSD-FD: 130.09 ± 12.41; HSD-FD: 129.86 ± 10.29), relative to that observed in the LSD-F group (83.58 ± 5.87). The telencephalic content of 5-HT and its main metabolite, 5HIAA (Figure 2B,D), did not display any significant variations among the experimental groups. The 5HIAA/5HT ratio (Figure 2F) was 33.18 ± 4.51 in the LSD-F group. A significant increase (*p* < 0.05) of the ratio was observed in the HSD-F group (54.04 ± 3.47), with respect to the LSD-F; such an increase did not reach a level of significance in those groups of animals subjected to the FD (LSD-FD: 44.49 ± 4.14; HSD-FD: 45.77 ± 4.84).

Finally, in the optic tectum (Figure 3), the DA and DOPAC contents and DOPAC/DA ratio (Figure 3A,C,E) did not display significant variations among the groups, similar to that for the 5HT and 5HIAA contents (Figure 3B,D). The 5HIAA/5HT (Figure 3F) ratio was 66.88 ± 9.18 in the LSD-F group. The HSD together with FD significantly enhanced this ratio (100.24 ± 4.63) with respect to the LSD-F group (*p* < 0.05). A similar trend was also observed in fish exposed to a HSD (83.37 ± 6.09) or FD (79.80 ± 3.42), relative to a LSD-F, but did not reach a level of significance.

## 4. Discussion

The growth models of gilthead sea bream for aquaculture have been reported (for review, see reference [35]). However, how different stressors influence sea bream growth do still need to be elucidated. In order to provide more information in this respect, the present study focused on the effect of a high stocking density (HSD) and food deprivation (FD) as potentially stressful situations that can be exposed when fish, including the gilthead sea bream, are produced in intensive culture conditions or even during overwintering when the water temperature drops in the environment [25]. Following exposure to stress, both the hypothalamus–sympathetic nervous system–chromaffin axis (HSC) and the hypothalamus–pituitary–inter-renal axis (HPI) activate important interconnected and endocrine-related cascades. As a consequence, a rapid increase of the plasma catecholamine and cortisol levels occurs, which results in multiple alterations at the metabolic and functional levels, thus affecting the overall fish physiology [1,2,3,5,6]. According to our results, a HSD and FD are effective in inducing chronic stress in sea bream, with the subsequent increase of the plasma cortisol in concordance with that previously reported for this species [26,27,28], which validates our results and the experimental approach performed.

When fish are subjected to a stressor, the HSC axis reacts to rapidly increase the plasma catecholamine (A and NA) levels but remains elevated for a short period of time [31]. Similar changes are reported for sea breams submitted to air exposure for 3 min [32]. In addition, the brain serotonergic activity is apparently involved in the HSC axis activation, leading to an increased release of A and NA into the blood [31,36]. Both catecholamines remain elevated, in parallel to the serotonergic activity. Thus, peripheral serotonin promotes the release of catecholamines in mammals and fish through a cholinergic innervation-independent mechanism [37,38,39]. Our results are consistent with such an idea, since fish kept under HSD and normal feeding conditions display an increase of the plasma A and NA levels, even when such an increase does not reach a level of significance. Such a result was also noted in food-deprived animals subjected to HSD but did not reach the magnitude on that observed in HSD-F sea breams. Then, it seems that food deprivation might lead to a decrease of the plasma catecholamine levels. Further research needs to be performed in order to corroborate this effect and the underlying mechanisms involved. Anyway, the brain monoaminergic activity may play a role in the maintenance of the physiological response and their homeostasis to moderate chronic stress in fish (see references [13,14,15,16,17,18,19,20] for review). 

Enhanced dopaminergic and serotonergic activities in response to stress exposure have been previously reported in several fish species [24,40,41]. The fact that different brain regions display such enhanced activities may evidence the involvement of brain DA and 5HT neurons in the primary response when the stress exposure occurs [21]. However, it is not clear whether or not monoaminergic systems participate in maintaining the endocrine response to stress in fish. In order to corroborate such a role, we also assessed the dopaminergic and serotonergic activities in different brain regions (the hypothalamus, telencephalon, and optic tectum) during chronic stress. We observe the altered contents of monoaminergic neurotransmitters, with the changes clearly dependent on the tissue assessed. Similar results were previously reported for rainbow trout, in which the increased content of the DA and 5HT metabolites (DOPAC and 5HIAA, respectively) occurred in the telencephalon, hypothalamus, preoptic region, brainstem, and optic tectum [14], with the subsequent increases of their respective metabolite/monoamine ratios. It is interesting to remark that changes of these ratios are considered as good indicators of monoaminergic activity enhancement during stress exposure [14,17,31,33,42,43] in a way that increases the values of a given ratio that occurs as a consequence of enhanced neuronal activity. Taking that into consideration, our results also agree with those previously reported for the brain monoamines contents of rainbow trout under different stress situations [20,44]. However, recent studies carried out in sea breams subjected to stress by 30 min of confinement every 48 h did not report any significant changes for 5HT, its metabolite (5HIAA), and the 5HIAA/5HT ratio in the brainstem [23]. This discrepancy might be due to the fact that a different stress protocol was performed, whereas our animals were chronically subjected to a HSD during the complete period that experiment lasted (21 days) instead of intermittent challenges. Additionally, the brainstem samples were evaluated, whereas our data came from the assessments of discrete brain regions (the telencephalon, hypothalamus, and optic tectum). Thus, the response to stress in fish does not only depend on the species but, also, on the stressor, the duration of exposure to stress, and brain area assessed.

Regarding the dopaminergic activity, our results demonstrate the hypothalamic DOPAC content and DOPAC/DA ratio enhancement, as well as a lowering effect in the telencephalic DA content, with a consequent increase in the DOPAC/DA ratio in animals subjected to each stressor alone or even when combined. On the contrary, such an increase was not observed in the optic tectum. These results are in agreement with that previously reported for other fish species, such as rainbow trout [14,33], Arctic charr [45], and Senegalese sole [21], submitted to different stress situations. Then, our results point out the role played by dopaminergic activity during the chronic stress response in sea breams and also suggest that this effect does not seem accumulative when both stressors are applied together. We also hypothesize that each of them alone is able to overload the endocrine cascade triggered at the central level.

With respect to the serotonergic activity, the 5HT and 5HIAA levels increased in the hypothalamus, and the 5HIAA/5HT ratio increased in different brain regions (the hypothalamus, telencephalon, and optic tectum). However, each brain region displayed a specific response to each stressor. Thus, both the HSD and FD challenges enhanced the serotonergic activity in the hypothalamus (both HSD groups) and optic tectum (HSD-FD group), whereas in the telencephalon, this activity was only increased in fish exposed to a HSD. Overall, our results agree with that previously reported for other fish species, such as Senegalese sole, in which the serotonergic activity was stimulated in the hypothalamus and telencephalon of animals subjected to a HSD but, also, to a low water renovation [21] or even in rainbow trout, showing that a HSD enhances the serotonergic activity in the hypothalamus, telencephalon, and optic tectum [14]. Then, the serotonergic activity also participates in the response to chronic stress in sea breams, similar to the dopaminergic activity.

Taken together, all the herein reported results are likely in agreement with the previous studies carried out regarding the effects of stress on the brain monoaminergic activities of fish [14,20,22,24,44]. Thus, considering the monoaminergic neuronal activity, changes in the DA and 5HT levels are indicative of the incidence of stress in sea breams in particular, and farmed fish in general, which results in jeopardized neurotransmitter functions. For both of them, the altered functions can be due to the increased neuronal turnover, in which the neurotransmitter is produced and released to the synaptic cleft but, also, reuptaken and degraded to the oxidative metabolite (DOPAC and 5HIAA) in the different brain regions. Whatever the case, the DOPAC/DA and 5HIAA/5HT ratios change, which is indicative of monoaminergic activity enhancement during chronic stress exposure in sea breams, in concordance with those changes observed for the plasma cortisol levels.

## 5. Conclusions

In summary, this study evidences that exposing sea breams to chronic stress by a high stocking density and/or food deprivation results in the enhancement of the stress response-related parameters (plasma cortisol levels and monoaminergic activities in different brain regions). As a consequence, their welfare is compromised. The aquaculture industry needs to fix this handicap. In order to help in dealing with this issue, we reported an enhanced monoaminergic activity (mainly in the hypothalamus and telencephalon) caused by exposure to stress. This result is indicative of the role played by monoaminergic systems not only in initiating the endocrine response to stress but, also, in maintaining it. Further research must be carried out in order to corroborate such an idea or even to avoid such fish reactions during the complete cycle of production by the use of nutraceutical compounds or feed supplementations and, also, to elucidate the underlying mechanisms through which this modulatory action takes place. This issue is very important for this economic activity, since it may be useful in developing strategies that help to minimize the impact of typical practices and stocking conditions in the aquaculture.

## Figures and Tables

**Table 1:** Plasma levels of cortisol, adrenaline, and noradrenaline of *S. aurata* juveniles subjected to feeding or food deprivation conditions, together with low or high stocking densities. * Significantly different (*p* < 0.05) at the same stocking density; # significantly different (*p* < 0.05) at the same feeding condition (*p* < 0.05, two-way ANOVA, followed by Tukey’s test). Values represent the average ± S.E.M.; *n* = 12 samples per group.

Parameter	Density	Fed	Food-Deprived
Cortisol	Low	4.17 ± 0.67	10.06 ± 2.68 *
	High	27.36 ± 6.82 #	23.87 ± 6.61 #
Adrenaline	Low	97.56 ± 10.05	80.85 ± 13.97
	High	148.74 ± 27.53	108.63 ± 35.57
Noradrenaline	Low	79.78 ± 6.72	49.03 ± 4.99 *
	High	91.88 ± 13.67	70.92 ± 24.62

## References

[1] Wendelaar Bonga S.E. (1997). The stress response in fish. Physiol. Rev..

[2] Schreck C.B., Tort L., Schreck C.B., Tort L., Farrell A.T., Brauner C.J. (2016). The concept of stress in fish. Biology of Stress in Fish.

[3] Mommsen T.P., Vijayan M.M., Moon T.W. (1999). Cortisol in teleosts: Dynamics, mechanisms of action, and metabolic regulation. Rev. Fish Biol. Fisher..

[4] Iwama G.K., Afonso L.O.B., Vijayan M.M., Evans D.H., Claiborne J.B. (2006). Stress in fishes. The Physiology of Fishes.

[5] Ellis T., Yildiz H.Y., López-Olmeda F.J., Spedicato M.T., Tort L., Øverli Ø., Martins C.I. (2012). Cortisol and finfish welfare. Fish Physiol. Biochem..

[6] Gorissen M., Flik G., Schreck C.B., Tort L., Farrell A.T., Brauner C.J. (2016). The Endocrinology of the Stress Response in Fish: An Adaptation-Physiological View. Biology of Stress in Fish.

[7] Flik G., Peter H.M., Klaren P.H.M., Erwin H., Van den Burg J.R., Huising M.O. (2006). CRF and stress in fish. Gen. Comp. Endocrinol..

[8] O’Connell L.A., Fontenot M.R., Hofman H.A. (2011). Characterization of the dopaminergic system in the brain of an african cichlid fish, *Astatotilapia burtoni*. J. Comp. Neurol..

[9] Yamamoto K., Ruuskanen J.O., Wullimann M.F., Vernier P. (2010). Two tyrosine hydroxylase genes in vertebrates: New dopaminergic territories revealed in the zebrafish brain. Mol. Cell. Neurosci..

[10] Moret F., Guilland J.C., Coudouel S., Rochette L., Vernier P. (2004). Distribution of tyrosine hyfroxilase, dopamine, and serotonin in the central nervous system of amphioxus (*Branchiostoma lanceolatum*): Implications for the evolution of catecholamine systems in vertebrates. J. Comp. Neurol..

[11] Lillesaar C. (2011). The serotonergic system in fish. J. Chem. Neuroanat..

[12] Pierre J., Repérant J., Ward R., Vesselkin N.P., Rio J.-P., Miceli D., Kratskin I. (1992). The serotoninergic system of the brain of the lamprey, *Lampetra fluviatilis*: An evolutionary perspective. J. Chem. Neuroanat..

[13] Øverli Ø., Winberg S., Pottinger T.G. (2005). Behavioral and neuroendocrine correlates of selection for stress responsiveness in rainbow trout-a review. Integr. Comp. Biol..

[14] Gesto M., Soengas J.L., Míguez J.M. (2008). Acute and prolonged stress responses of brain monoaminergic activity and plasma cortisol levels in rainbow trout are modified by PAHs (naphthalene, β-naphthoflavone and benzo(a)pyrene) treatment. Aquat. Toxicol..

[15] Winberg S., Lepage O. (1998). Elevation of brain 5-HT activity, POMC expression, and plasma cortisol in socially subordinate rainbow trout. Am. J. Physiol. Reg. Int. Comp. Physiol..

[16] Höglund E., Weltzien F.A., Schjolden J., Winberg S., Ursin H., Døving K.B. (2005). Avoidance behavior and brain monoamines in fish. Brain Res..

[17] Ruibal C., Soengas J.L., Aldegunde M. (2002). Brain serotonin and the control of food intake in rainbow trout (*Oncorhynchus mykiss*): Effects of changes in plasma glucose levels. J. Comp. Physiol. A.

[18] De Pedro N., Delgado M.J., Gancedo B., Alonso-Bedate M. (2003). Changes in glucose, glycogen, thyroid activity and hypothalamic catecholamines in tench by starvation and refeeding. J. Comp. Physiol..

[19] Øverli Ø., Harris C.A., Winberg S. (1999). Short-term effects of fights for social dominance and the establishment of dominant-subordinate relationships on brain monoamines and cortisol in rainbow trout. Brain Behav. Evol..

[20] Øverli Ø., Pottinger T.G., Carrick T.R., Øverli E., Winberg S. (2001). Brain monoaminergic activity in rainbow trout selected for high and low stress responsiveness. Brain Behav. Evol..

[21] López-Patiño M.A., Conde-Sieira M., Gesto M., Librán-Pérez M., Soengas J.L., Míguez J.M. (2013). Melatonin partially minimizes the adverse stress effects in Senegalese sole (*Solea senegalensis*). Aquaculture.

[22] Conde-Sieira M., Muñoz J.L.P., López-Patiño M.A., Gesto M., Soengas J.L., Míguez J.M. (2014). Oral administration of melatonin counteracts several of the effects of chronic stress in rainbow trout. Domest. Anim. Endocrinol..

[23] Samaras A., Espírito Santo C., Papandroulakis N., Mitrizakis N., Pavlidis M., Höglund E., Pelgrim T.N.M., Zethof J., Spanings F.A.T., Vindas M.A. (2018). Allostatic load and stress physiology in European seabass (*Dicentrarchus labrax* L.) and gilthead Seabream (*Sparus aurata* L.). Front. Endocrinol..

[24] Herrera M., Fernández-Alacid L., Sanahuja I., Ibarz A., Salamanca N., Morales E., Giráldez I. (2020). Physiological and metabolic effects of a tryptophan-enriched diet to face up chronic stress in meagre (*Argyrosomus regius*). Aquaculture.

[25] Pavlidis M., Mylonas C. (2011). Sparidae: Biology and Aquaculture of Gilthead Sea Bream and Other Species.

[26] Mancera J.M., Vargas-Chacoff L., García-López A., Kleszczyńska A., Kalamarz H., Martínez-Rodríguez G., Kulczykowska E. (2008). High density and food deprivation affect arginine vasotocin, isotocin and melatonin in gilthead sea bream (*Sparus auratus*). Comp. Biochem. Physiol. A Mol. Integr. Physiol..

[27] Skrzynska A.K., Martos-Sitcha J.A., Martínez-Rodríguez G., Mancera J.M. (2018). Unraveling vasotocinergic, isotocinergic and stress pathways after food deprivation and high stocking density in the gilthead sea bream. Comp. Biochem. Physiol. A Mol. Integr. Physiol..

[28] Arends R.J., Mancera J.M., Muñoz J.L., Wendelaar Bonga S.E., Flik G. (1999). The stress response of the gilthead sea bream (*Sparus aurata* L.) to air exposure and confinement. J. Endocrinol..

[29] Sangiao-Alvarellos S., Guzmán J.M., Láiz-Carrión R., Míguez J.M., Martín del Río M.P., Mancera J.M., Soengas J.L. (2005). Interactive effects of high stocking density and food-deprivation on carbohydrate metabolism in several tissues of gilthead sea bream, *Sparus aurata*. J. Exp. Zool..

[30] Martos-Sitcha J.A., Wunderink Y.S., Straatjes J., Skrzynska A.K., Mancera J.M., Martínez-Rodríguez G. (2014). Different stressors induce differential responses of the CRH-stress system in the gilthead sea bream (*Sparus aurata*). Comp. Biochem. Physiol. A Mol. Integr. Physiol..

[31] Gesto M., López-Patiño M.A., Hernández J., Soengas J.L., Míguez J.M. (2013). The response of brain serotonergic and dopaminergic systems to an acute stressor in rainbow trout: A time course study. J. Exp. Biol..

[32] Skrzynska A.K., Maiorano E., Bastaroli M., Naderi F., Míguez J.M., Martínez-Rodríguez G., Mancera J.M., Martos-Sitcha J.A. (2018). Impact of air exposure on vasotocinergic and isotocinergic systems in gilthead sea bream (*Sparus aurata*): New insights on fish stress response. Front. Physiol..

[33] Gesto M., Tintos A., Soengas J.L., Míguez J.M. (2006). Effects of acute and prolonged naphthalene exposure on brain monoaminergic neurotransmitters in rainbow trout (*Oncorhynchus mykiss*). Comp. Biochem. Physiol..

[34] Smith P.K., Krohn R.I., Hermanson G.T., Mallia A.K., Gartner F.H., Provenzano M.D. (1985). Measurement of protein using bicinchoninic acid. Anal. Biochem..

[35] Seginer I. (2016). Growth models of gilthead sea bream (*Sparus aurata* L.) for aquaculture: A review. Aquacult. Eng..

[36] Gesto M., Otero-Rodiño C., López-Patiño M.A., Míguez J.M., Soengas J.L., Conde-Sieira M. (2014). Is plasma cortisol response to stress in rainbow trout regulated by catecholamine-induced hyperglycemia?. Gen. Comp. Endocrinol..

[37] Winberg S., Nilsson A., Hylland P., Söderstöm V., Nilsson G.E. (1997). Serotonin as a regulator of hypothalamic-pituitary-interrenal activity in teleost fish. Neurosci. Lett..

[38] Reid S.G., Bernier N.J., Perry S.F. (1998). The adrenergic stress response in fish: Control of catecholamine storage and release. Comp. Biochem. Physiol..

[39] Fabbri E., Moon T.W. (2016). Adrenergic signaling in teleost fish liver, a challenging path. Comp. Biochem. Physiol. B Biochem. Mol. Biol..

[40] Atwood H.L., Tomasso J.R., Ronan P.J., Barton B.A., Renner K.J. (2000). Brain monoamine concentrations as predictors of growth inhibition in channel catfish exposed to ammonia. J. Aquat. Anim. Health.

[41] Ortega V.A., Renner K.J., Bernier N.J. (2005). Appetite-suppressing effects of ammonia exposure in rainbow trout associated with regional and temporal activation of brain monoaminergic and CRF systems. J. Exp. Biol..

[42] Aldegunde M., Mancebo M. (2006). Effect of neuropeptide Y on food intake and brain biogenic amines in the rainbow trout (*Oncorhynchus mykiss*). Peptides.

[43] Soengas J.L., Aldegunde M. (2004). Brain glucose and insulin: Effects on food intake and brain biogenic amines of rainbow trout. J. Comp. Physiol. A.

[44] Lepage O., Tottmar O., Winberg S. (2002). Elevated dietary intake of l-tryptophan counteracts the stress-induced elevation of plasma cortisol in rainbow trout (*Oncorhynchus mykiss*). J. Exp. Biol..

[45] Höglund E., Balm P.H.M., Winberg S. (2000). Skin darkening, a potential social signal insubordinate Arctic charr (*Salvelinus alpinus*): The regulatory role of brain monoaminesand pro-opiomelanocortin-derived peptides. J. Exp. Biol..

